# Retroperitoneal Hibernoma Presenting as a Gastrointestinal Surgical Diagnostic Challenge: A Rare Benign Lipomatous Tumor and Literature Review

**DOI:** 10.1155/crgm/7500074

**Published:** 2026-10-04

**Authors:** Gilton Marques Fonseca, Luiz Guilherme Cernaglia Aureliano de Lima, Marcel Cerqueira Cesar Machado

**Affiliations:** ^1^ Liver Surgery Unit Department of Gastroenterology University of Sao Paulo Medical School Sao Paulo Brazil usp.br; ^2^ Department of Pathology Hospital Sirio-Libanes Sao Paulo Brazil; ^3^ Department of Clinical Medicine University of Sao Paulo Medical School Sao Paulo Brazil usp.br

**Keywords:** atypical lipoma, fatty tumor, hibernoma, lipomatous neoplasm, retroperitoneal tumor

## Abstract

**Background:**

Hibernoma is an uncommon benign adipocytic neoplasm showing differentiation toward brown fat. Although it usually occurs in anatomical sites where residual brown adipose tissue persists, retroperitoneal involvement is particularly uncommon, with fewer than 60 cases reported.

**Case Presentation:**

A 54‐year‐old man was found to have a retroperitoneal mass incidentally during a routine physical examination. CT and MRI demonstrated a 15‐cm homogeneous fat‐containing lesion without radiological evidence of local invasion. Complete surgical excision was performed. Histopathological examination showed univacuolated and multivacuolated adipocytic cells without atypia. The tumor was positive for S‐100 and CD10, and fluorescence in situ hybridization showed no MDM2 amplification, supporting the diagnosis of hibernoma. Recovery was uneventful, and the patient was discharged on Postoperative Day 7.

**Discussion:**

Retroperitoneal hibernoma can be difficult to distinguish radiologically from other lipomatous neoplasms, including atypical lipomatous tumor/well‐differentiated liposarcoma (ALT/WDL). Diagnosis therefore relies on the integration of morphology with ancillary immunohistochemical and, when indicated, molecular findings. The decision between preoperative core needle biopsy and upfront resection should be individualized according to the radiological differential diagnosis, tumor vascularity and resectability, expected surgical morbidity, and whether tissue confirmation would alter management.

**Conclusion:**

Hibernoma should be included in the differential diagnosis of retroperitoneal fat‐containing masses. Accurate diagnosis is fundamental, as complete surgical excision is curative, with no need for additional treatment.

## 1. Introduction

Hibernoma is a benign adipocytic neoplasm composed of cells that recapitulate brown adipose tissues. The lesion was initially described by Merkel in 1906 as a “pseudolipoma” and was subsequently termed hibernoma by Gery in 1914 [1]. It represents only a small proportion of adult adipocytic tumors and is most often diagnosed in young to middle‐aged adults, without a consistent sex predominance [2–4]. Common sites include the neck, axilla, thorax, and extremities, which correspond to regions in which remnants of brown fat may persist. In contrast, retroperitoneal hibernoma is distinctly uncommon [5, 6]. Because a deep retroperitoneal fat‐containing mass may resemble other benign or malignant lipomatous tumors on imaging, establishing the correct diagnosis has direct implications for management.

## 2. Case Presentation

A 54‐year‐old man with diabetes mellitus and dyslipidemia was found to have a fixed left‐sided abdominal mass during a routine examination. Abdominal CT and MRI demonstrated a 15‐cm homogeneous retroperitoneal mass with predominantly lipomatous characteristics and no radiological evidence of local invasion (Figure 1). Cholelithiasis was also identified. Based on the imaging findings, retroperitoneal lipoma was the leading preoperative diagnosis, although other fat‐containing neoplasms, particularly atypical lipomatous tumor/well‐differentiated liposarcoma (ALT/WDL), were included in the differential diagnosis. Because the lesion appeared technically resectable and showed no evidence of local invasion, upfront surgical resection was selected. The patient underwent open resection of the retroperitoneal mass together with cholecystectomy. Gross examination demonstrated a well‐circumscribed, encapsulated mass measuring 15.3 × 12.6 × 5.1 cm and weighing 680 g (Figure 2).

**FIGURE 1:**
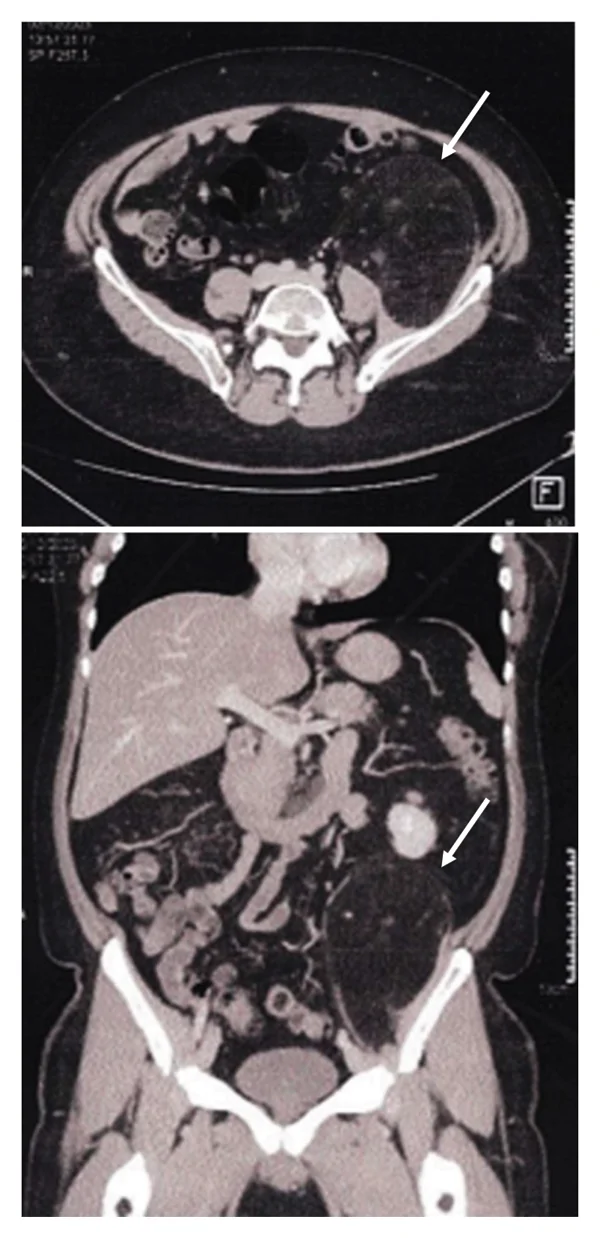
Contrast‐enhanced computed tomography of the abdomen. (A) Axial image showing a large low‐attenuation, fat‐containing mass in the left retroperitoneum, with displacement of adjacent structures; the arrow identifies the lesion. (B) Coronal image demonstrating the craniocaudal extent of the left retroperitoneal mass and its relationship to adjacent abdominal structures; the arrow identifies the lesion.

**FIGURE 2:**
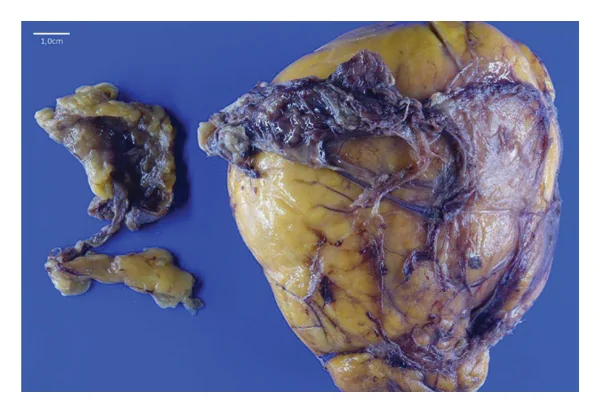
Gross appearance of the resected retroperitoneal tumor. The specimen is a well‐circumscribed, encapsulated, yellow lobulated mass measuring 15.3 × 12.6 × 5.1 cm, with brown fibrous bands visible on its surface.

Histopathological examination showed an adipocytic proliferation composed predominantly of multivacuolated brown fat‐like cells separated by delicate fibrous septa (Figure 3). Immunohistochemical staining demonstrated strong expression of S‐100 and CD10. Fluorescence in situ hybridization (FISH) showed no MDM2 amplification (Figure 4). Taken together with the morphological findings, these results supported the diagnosis of hibernoma. The postoperative course was uncomplicated, and the patient was discharged on Postoperative Day 7.

**FIGURE 3:**
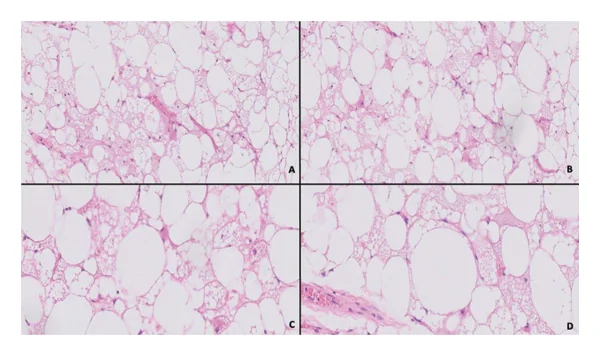
Histological features of the tumor on hematoxylin and eosin staining (200 ×). (A–D) An admixture of univacuolated, paucivacuolated, and multivacuolated adipocytic cells, reflecting white and brown fat differentiation. The cells have small, normochromatic nuclei without cytological atypia.

**FIGURE 4:**
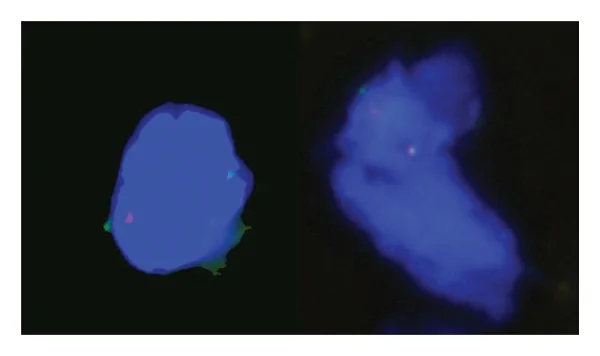
Fluorescence in situ hybridization (FISH) with DAPI nuclear counterstaining (1000 ×). No MDM2 amplification is identified. In the context of the histomorphological and immunohistochemical findings, the absence of MDM2 amplification supports hibernoma over atypical lipomatous tumor/well‐differentiated liposarcoma.

## 3. Discussion

The pathological appearance of hibernoma reflects brown fat differentiation. Tumor cells may contain finely vacuolated or granular eosinophilic cytoplasm, related to their abundant mitochondrial content, and the lesions are often conspicuously vascular [2]. Although the amount of brown adipose tissue decreases markedly after infancy, residual deposits remain in adults [7]. The indolent growth and deep location of retroperitoneal lesions may allow them to become large before they are detected [2].

The principal differential diagnoses of a retroperitoneal fat‐containing mass include conventional lipoma and ALT/WDL; other adipocytic lesions may also enter the differential depending on patient age and morphology [8]. Cross‐sectional imaging can define the extent, composition, and relationship of the tumor to adjacent structures, but imaging findings alone may not reliably establish the diagnosis [1]. Histomorphology remains central to diagnosis, while ancillary studies can be useful in difficult cases. S‐100 and CD10 expressions may support brown fat differentiation, whereas assessment of MDM2 amplification is particularly helpful when ALT/WDL is a concern [9–11].

CD10 expression has been reported as a potentially useful adjunct in distinguishing hibernoma from morphologic mimics [11]. At the molecular level, hibernomas characteristically harbor structural abnormalities involving Chromosome 11q13, a region that includes MEN1 and AIP [9, 10]. Hibernomas have also been described in association with multiple endocrine neoplasia Type 1, although this association is uncommon [12]. These findings underscore the value of integrating morphology, immunophenotype, and molecular testing rather than relying on a single marker.

For a resectable lesion in which surgery is already indicated and can be performed with acceptable morbidity, upfront complete excision may provide both definitive diagnosis and treatment. Conversely, when surgery would entail substantial morbidity or when histopathological confirmation would materially influence the therapeutic strategy, particularly when liposarcoma is suspected, image‐guided core needle biopsy should be considered after careful assessment of tumor vascularity, biopsy feasibility, and procedural risk. Therefore, the choice between preoperative biopsy and upfront resection should be individualized according to the radiological differential diagnosis, tumor vascularity and resectability, anticipated surgical morbidity, and whether a preoperative tissue diagnosis would alter management.

Previously reported retroperitoneal and intra‐abdominal hibernomas have usually been described as isolated case reports or small case series, reflecting the rarity of this location [2, 10–13]. Reported sites include the retroperitoneum, kidney/perirenal region, presacral space, and extraperitoneal pelvis, with tumors often discovered incidentally or after nonspecific abdominal symptoms related to the mass effect. In most cases, imaging demonstrated a well‐circumscribed fat‐containing mass, but preoperative distinction from other lipomatous tumors, particularly ALT/WDL, was challenging. As in the present case, definitive diagnosis generally depended on the histopathological examination supported by immunohistochemistry and, when necessary, molecular testing. For confirmed hibernoma, complete surgical excision is generally curative [14]. Published series and case reports have not demonstrated metastatic behavior, and recurrence after complete excision is exceptionally uncommon [1, 13]. This favorable biological behavior makes accurate distinction from ALT/WDL and other malignant retroperitoneal tumors clinically important.

From a gastrointestinal surgical perspective, this case highlights the diagnostic and management challenges posed by deep retroperitoneal fat‐containing masses. Although hibernoma is benign, its retroperitoneal location, large size, and lipomatous appearance may closely mimic other tumors more commonly considered in gastrointestinal and abdominal surgical practice, particularly ALT/WDL. For gastroenterologists and gastrointestinal surgeons, the key clinical issue is not only to recognize hibernoma as a rare differential diagnosis but also to determine whether preoperative tissue diagnosis is necessary before resection. In technically resectable lesions without radiological signs of invasion and with acceptable operative morbidity, upfront excision may provide both diagnosis and definitive treatment. Conversely, when imaging suggests liposarcoma, when resection would require major multivisceral surgery, or when histological confirmation would alter the therapeutic strategy, image‐guided core needle biopsy should be considered. The main clinical message is that retroperitoneal hibernoma, although rare, should be remembered in the differential diagnosis of fat‐containing abdominal masses because accurate distinction from ALT/WDL avoids overtreatment, and complete excision is curative.

## 4. Conclusion

Retroperitoneal hibernoma is a rare benign adipocytic neoplasm that may resemble other fat‐containing retroperitoneal tumors on imaging. Definitive diagnosis depends on histomorphological assessment, supported when appropriate by immunohistochemical and molecular studies; the absence of MDM2 amplification is particularly useful when ALT/WDL is in the differential diagnosis. Management should be individualized according to resectability, procedural risk, and whether preoperative tissue confirmation would alter treatment. Recognition of hibernoma is important because complete excision is curative, and unnecessary additional oncological treatment can be avoided.

## Author Contributions

Gilton Marques Fonseca and Luiz Guilherme Cernaglia Aureliano de Lima contributed to the writing of the manuscript. Luiz Guilherme Cernaglia Aureliano de Lima performed the pathological evaluation. Marcel Cerqueira Cesar Machado critically reviewed and revised the final version of the manuscript.

## Funding

No funding was received for this study.

## Consent

The patient provided informed consent for the publication of this case report.

## Conflicts of Interest

The authors declare no conflicts of interest.

## Data Availability

The data that support the findings of this study are available on request from the corresponding author. The data are not publicly available due to privacy or ethical restrictions.
